# Crude Citric Acid of *Trichoderma asperellum*: Tomato Growth Promotor and Suppressor of *Fusarium oxysporum* f. sp. *lycopersici*

**DOI:** 10.3390/plants10020222

**Published:** 2021-01-24

**Authors:** Abdulaziz A. Al-Askar, WesamEldin I. A. Saber, Khalid M. Ghoneem, Elsayed E. Hafez, Amira A. Ibrahim

**Affiliations:** 1 Botany and Microbiology Department, Faculty of Science, King Saud University, Riyadh 11451, Saudi Arabia; aalaskara@ksu.edu.sa; 2 Microbial Activity Unit,Microbiology Department, Soils, Water and Environment Research Institute, Agricultural Research Center (ID: 60019332), Giza 12112, Egypt; 3 Seed Pathology Research Department, Plant Pathology Research Institute, Agricultural Research Center (ID: 60019332), Giza 12112, Egypt; khalid_ghoneem@yahoo.com; 4 Plant Protection and Biomolecular Diagnosis Department, Arid Lands Cultivation Research Institute, City of Scientific Research and Technological Applications, New Borg El-Arab, Alexandria 21934, Egypt; elsayed_hafez@yahoo.com

**Keywords:** biological control, Fusarium wilt disease, molecular identification, organic acids

## Abstract

Presently, the bioprocessing of agricultural residues to various bioactive compounds is of great concern, with the potential to be used as plant growth promoters and as a reductive of various diseases. *Lycopersicon* *esculentum*, one of the most consumed crops in the human diet, is attacked by Fusarium wilt disease, so the main aim is to biocontrol the pathogen. Several fungal species were isolated from decayed maize stover (MS). *Trichoderma* *asperellum* was chosen based on its organic acid productivity and was molecularly identified (GenBank accession number is MW195019). Citric acid (CA) was the major detected organic acid by HPLC. In vitro, CA of *T.* *asperellum* at 75% completely repressed the growth of *Fusarium* *oxysporum* f. sp. *lycopersici* (FOL). In vivo, soaking tomato seeds in CA enhanced the seed germination and vigor index. *T. asperellum* and/or its CA suppressed the wilt disease caused by FOL compared to control. There was a proportional increment of plant growth and yield, as well as improvements in the biochemical parameters (chlorophyll pigments, total phenolic contents and peroxidase, and polyphenol oxidase activities), suggesting targeting both the bioconversion of MS into CA and biological control of FOL.

## 1. Introduction

Tomato (*Lycopersicon esculentum* Mill.) is one of the most significant vegetable crops grown in the world. It is a major contributor to the fruit and vegetable diet of humans, representing an excellent source of various micronutrients and antioxidants for controlling cholesterol and weight reduction [1]. In Sauda Arabia, the cultivated area for this crop is around 13,055 ha, yielding around 332,608 tons with productivity of 25.48 t/ha [2].

The tomato plant is infected by diverse destructive diseases that drastically influence its growth and yield. Among them, Fusarium wilt disease, caused by *Fusarium oxysporum* Schlechtend: Fr. f. sp. *lycopersici* (Sacc.) W.C. Snyder and H.N. Hansen, is the limiting factor and widespread disease of the cultivated tomato. The pathogen occurs and devastates tomatoes in most growing regions, resulting in a yield loss of up to 66% in Saudi Arabia [3], especially on the susceptible varieties, when soil and air temperature are rather high during the warm season [4]. The virulence profile of *F. oxysporum* f. sp. *lycopersici* isolates influencing tomatoes has been grouped into three races according to their capability to infect a varying set of cultivars, having distinct resistance loci. Races 1 and 2 are circulated worldwide, whereas race 3 has a narrow geographic distribution [5].

In other words, resistant varieties have been used as the most effective strategy to control the disease, but it can be weakened by new races of the pathogen, which emerge in response to the newly resistant varieties [5,6]. Additionally, another strategy applied to control these fungal diseases, namely the use of agrochemicals, is an effective procedure to eradicate and control phytopathogens. However, the excessive and long-term application of agrochemicals can cause environmental and health problems, and such agrochemicals could generate a new resistant strain of fungal pathogens [7].

Therefore, biological control is considered one of the effective strategies to fight soil-borne fungal pathogens, since several investigators have reported *Trichoderma* spp. as biological control agents against many phytopathogenic fungi, i.e., *Fusarium* species [8,9,10]. Variation in antagonistic mechanisms has been proposed, including direct and/or indirect mechanisms. Direct mechanisms include microbial competition for space and/or nutrients, antibiosis, and mycoparasitism, while indirect mechanisms include induction of defense responses in the host plant and/or plant growth enhancement to increase yields [11]. Moreover, *Trichoderma* spp. are known to produce various bioactive secondary metabolites that promote plant growth such as indole-3-acetic acid [9,12]. Moreover, *Trichoderma asperellum* Samuels Lieckf. and Nirenberg is a mycoparasitic species that is well known and widely used for its ability to control several plant pathogens including the Fusarium wilt pathogen of tomato control systems [8,13].

A number of other soil-borne pathogens, e.g., *F. oxysporum* f. sp. *asparagi*, *Rhizoctonia solani*, and *Verticillium dahlia,* can be repressed under anaerobic conditions, and this suppression may be enhanced during the fermentation and decomposition of organic matter, which is followed by the development of organic acids (OA) formation [14,15]. In this respect, Momma [16] investigated the suppression of *F. oxysporum* f. sp. *lycopersici* and *Ralstonia solanacearum* during bio-hydrolysis of some organic matters and the production of acetic acid and/or butyric acid. The production of organic acid during bio-hydrolysis is another effective mechanism since the studies pointed out that OA application on seed-borne diseases played a crucial role in the inhibition of associated fungi, e.g., *Aspergillus fumigatus*, *A*. *nodulans*, *Penicillium roqueforti*, *Mucor* spp., and *Fusarium* spp. [17]. Another study revealed that OA (malic, citric, oxalic, and acetic) inhibited the growth of the phytopathogenic fungus, *Colletotrichum* sp., and a higher concentration of the acids was more efficient in reducing the fungal growth [18]. The kinetic suppression of microbial growth could be due to the disruption of the plasmic membrane that neutralizes its electrochemical potential and increases its permeability, and it could also reduce cytoplasmic pH and metabolic activities [19]. The suppression effect of some OA on *Aspergillus flavus*, *Penicillium purpurogenium*, *Rhizopus nigricans*, and *Fusarium oxysporum* was found to be extended to their mycotoxins production [20].

The present investigation trial dealt with the suppression of tomato wilt disease by *Trichoderma asperellum* and/or some of its OA (produced during the fermentation process on corn stover). The investigation was conducted both in vitro and in vivo on *F*. *oxysporum* f. sp. *lycopersici* ATCC^®^ 201829TM. Their effects on growth, physiological parameters, and yield were determined.

## 2. Materials and Methods

### 2.1. Fermentation Materials

A bulk of 19 samples of maize stover (MS) residuals were collected from Riyadh, Al-Ahsaa, Tabuk, and Al-Qaseem governorates, Saudi Arabia. Samples were dried (50 °C) overnight, then blended using an electric grinder to get a fine powder (maximum 0.1 cm long). The latter was served as solid-medium support and fermentable-substrate for organic acid production. The blended MS was not subjected to any pretreatment in order to simulate the natural growth environments of the microbes. The complex phosphorus compound, tricalcium phosphate (TCP) (Ca_3_(PO_4_)_2_) (Sigma-Aldrich, Saint Louis, MO, USA), was applied in the medium as a sole external complex inorganic phosphorus source.

### 2.2. Isolation of Cellulolytic Fungi

Cellulose-decomposing fungi were isolated from seven decayed samples of the collected MS. A weight of 500 g was placed in a clean plastic bag, labeled, and transferred to the laboratory for fungal isolation. The decayed samples of MS were homogenized well and cut into small pieces to obtain about 0.5 cm long pieces, and 5 pieces were placed in sterile Petri-plate lined with sterile filter papers wetted with sterile H_2_O. The plates were kept at 25 °C for 5 days with an alternative cycle of 12 h light and 12 h darkness using cool white fluorescent light. The growing fungal colonies were tested, using stereo and compound microscopes, to identify the developed fungi. Individual pure colonies of the grown fungi were obtained using the Hyphal-tip and/or single spore isolation techniques. The pure fungal cultures were conserved on slants of potato dextrose agar (PDA) for confirming the morphological identification and for performing further studies.

The morphological characterization was done, first to the genus only, and then full identification was carried out up to the species level, on the selected fungus, by observing the growth character of the fungus on agar plates. The measurements and examinations of the morphological structures and vegetative mycelia were investigated under a light microscope by mounting portions of fungal growth in a lactophenol cotton blue stain on clean slides [21,22,23].

All the obtained isolates were descriptively screened for cellulolytic activity by growing on plates of agar medium containing 0.5% carboxymethyl cellulose (CMC) at 25 °C and examined daily for up to 5 days. Active cellulolytic isolates were determined by the formation of a clear zone after flooding with 0.2% Congo red for 15 min, then destained by washing twice by 1 M NaCl for 15 min [24].

### 2.3. Culturing Conditions

The technique of solid-state fermentation (SSF) was used for screening the isolated fungal species their capabilities to produce organic acid. MS-based medium (1.0 g) was used to mimic the natural environmental growth conditions, in the presence of 15 mg TCP/g MS. The contents were mixed exhaustively with 5 mL tap water (pH 5) in 250 mL Erlenmeyer flasks and autoclaved (121 °C) for 15 min.

After cooling, flasks were injected with 1.0 mL of the tested fungal spores (1 × 10^8^/mL), prepared from a 7-days old culture. The moisture was kept constant by the addition of sterilized water when needed. After a period of 7-days incubation at 28 °C, 10 mL of 0.01% Tween 80 was added to the fermented constituents and shaken for 30 min on a rotary shaker (150 rpm). The fungal filtrate was then attained through separation by filter paper, which further centrifuged (5000 rpm for 20 min) for getting clear filtrate. The resulting post-culture filtrate was examined for the total organic acid content.

### 2.4. Quantification of Organic Acid (OA)

#### 2.4.1. Total OA Determination

The colorimetrical determination of the total OA in the filtrate was performed based on the method described by [25].

#### 2.4.2. Quantitation of OA using HPLC

The total OA in the post-culture filtrate was eluted by mixing a 1.0 g sample with methanol (20 mL) at 40 °C, then filtered and concentrated under reduced pressure to dryness (40 °C). The resultant concentrate was redissolved in acidulated water (pH2, with HCl), followed by evaporation at 40 °C under reduced pressure to dryness. The resultant concentrate was re-dissolved again in 1.0 mL of 0.01 N H_2_SO_4_. A volume of 20 µL was inspected of OA by HPLC unit (Agilent 1200 Infinity Series, St Albans, Hertfordshire, United Kingdom). A C18 column was used at 30 °C. Elution was carried out isocratically with 0.01 N H_2_SO_4_, as the mobile phase, at a flow rate of 0.1 mL/min, for 120 min. The UV detection was at 214 nm. The OA concentrations were determined using the peak areas and retention times of the OA standards [26].

### 2.5. Molecular Identification

The selected fungus was molecularly identified, applying the molecular biological protocol of DNA isolation and amplification via the polymerase chain reaction. For PCR amplification and sequencing of the internal transcribed spacer (ITS), the reaction mixture consisted of 1× buffer (Promega), 15 mM MgCl_2_, 0.2 mM dNTPs, 20-picomole of each primer (ITS1 (TCTGTAGGTGAACCTGCGG) and ITS4 (TCCTCCGCTTATTGATATGC)), 1 µL of Taq DNA polymerase (GoTaq, Promega), 40 ng DNA and ultra-pure water was added to a final total volume of 50 µL. Purification was accomplished to eliminate unincorporated PCR primers and dNTPs from PCR products using the Montage PCR Clean-up kit (Millipore). Sequencing was performed, using Big Dye terminator cycle sequencing kit (Applied BioSystems, USA) and then determined on an Applied Biosystems model 3730XL automated DNA sequencing system (Applied BioSystems, McCormick, SC, USA). The sequences were explored using the BLAST program (http://www.ncbi.nlm.nih.gov/BLAST) and aligned using Align Sequences Nucleotide BLAST. The software package; MEGA 10 was used for multiple alignment and phylogenetic analysis. The obtain sequence (600 bp) has been deposited in GenBank to obtain the closely related fungi sequences; then, the accession number of the fungal strain was received.

### 2.6. The Potential Antagonism of Trichoderma Filtrate

The antifungal behavior of the *Trichoderma* CA versus *F. oxysporum* f. sp. *lycopersici* ATCC*^®^* 201829TM race 2 (FOL) (American Type Culture Collection, Manassas, VA 20110, USA) was evaluated. The CA was mixed with sterilized PDA before solidification in conical flasks to obtain the concentrations of 15, 30, 45, 60, 75, and 90%. Twenty-milliliter portions of amended media were poured into 9 cm diameter Petri dishes, and another set of control PDA plates served as a control. All plates were inoculated, individually, with 0.5-cm diameter discs of *F. oxysporum* and then incubated in the dark (25 ± 2 °C) until the pathogen of control plates reached full growth.

### 2.7. Crude Citric Acid (CA) Against Seed Germination

The influence of CA on the germination and vigor index of tomato seeds (Farah variety), a common FOL-susceptible greenhouse cultivar to Fusarium wilt in Saudi Arabia, was investigated. Seed lot, with the lowest germination (~75%) and a high infection by fungal pathogens, was selected. The seeds were soaked in the bio-prepared CA for 2 h. The crop seeds were incubated for 14 days at 20 °C (16/8 h photoperiod), in a germination chamber. The germination test was conducted on 400 seeds using the between-paper method [27]. The vigor index was assessed using the following formula [28]:

Vigor index=DI (%) = Root length(cm)+Shoot length(cm))× Percentages of seed germination

Other disinfected seeds were treated with a recommended fungicide (Pink-S (5-methylisoxazol-3-ol) at 30% SL) for 1 h and used as a positive control. A set of untreated seeds was used as a negative control. The final count of germination and the measurement of the root and shoot lengths were estimated on the 14th day.

### 2.8. Evaluation of Crude CA under Greenhouse

Under greenhouse conditions, the application of *T. asperellum* was evaluated versus tomato wilt disease caused by FOL. The pathogenic fungus was grown in a bottle containing sterilized Sorghum: Coarse sand: Water (2:1:2, v/v) medium and incubated at 25 ± 2 °C for 14 days. Pots (40 cm diameter) filled with sterilized soil were used. The upper layer of the soil was infested with the inoculum of the pathogen at 0.4% kg soil^−1^. Tomato seedlings at the 3rd and 4th true leaf stage were surface-sterilized using sodium hypochlorite (1%), washed with sterile water, and then dried on filter paper. The seedlings were soaked in *T. asperellum* spore suspension (6 × 10^6^ spore mL^−1^) (Ts) or its CA for 1 h. Two seedlings per pot (40 cm diam.) were used with twenty replicates per treatment. A set of disinfected seedlings was dressed with the fungicide, Doviex SC 50% (Azoxystrobin 28.5% + Metalaxyl M 10.8%) as a positive control. Other untreated seedlings were used as a negative control.

For comparison, additional treatment was added, in which a set of tomato seedlings were soaked in a mixture solution of chemical citric acid (CCA) containing the same detected kinds and concentrations of OAs as was found in the filtrate obtained from HPLC analysis of *Trichoderma* filtrate. The pots were watered when necessary. A complete randomized block design was applied to arrange the experiment. Three blocks and 6 replicates for each treatment within the block were used.

#### 2.8.1. Wilt Disease Parameters

Wilt disease severity (DS) was assessed two weeks after transplanting on a scale of zero to 5 degrees according to [29], where 0 = no symptoms (neither root discoloration nor leaf yellowing), 1 = more than 0 up to 25% root discoloration or one leaf yellowed, 2 = more than 25 up to 50% root discoloration or more than one leaf yellowed, 3 = more than 50 up to 75% root discoloration or vascular discoloration plus one leaf wilted, 4 = more than 76% root discoloration or more than one leaf wilted and 5 = completely dead plant. The disease incidence (DI) was determined using the following formula [30].

DI (%) = (number of infected plants/ total number of plants)×100

#### 2.8.2. Investigated Parameters

After 20 days from transplanting, extraction, and activity of both polyphenol oxidase (PPO) and peroxidase (POD), enzymes were determined using a spectrophotometric method according to [31]. Total phenolic contents of fresh leaves were determined by using the Folin-Ciocalteu reagent method [32]. At 30 days after transplanting, chlorophyll (a, b, and total) and carotene contents in tomato leaves were determined [33].

Forty-five days after the transplantation, 6 plants of each trial were carefully pulled out with the entire root system and washed. The growth parameters (plant height, fresh and dry weights, and number of leaves) were measured. At physiological maturity (the time when dry matter accumulation in the fruits ceases, approximately 60 days after transplanting), fruits of each pot (40-cm diam.) were harvested and weighed separately to determine fruit yield.

### 2.9. Statistical Analysis

The statistical analysis software CoStat version 6.4 (CoHort Software, Pacific Grove, CA, USA) was used for the analysis of variance of the data, including wilt disease parameters (after transformation of the data according to the scale (DS) or equation (DI) above). Duncan’s new multiple range test at probability (*p*) level ≤ 0.05 was applied. This test can perform all possible pairwise comparisons among means, and there were larger differences between means, which guards against Type I error. Experimental data were presented as means ± standard deviation (±SD). At least six replicates were used in the experiments.

## 3. Results

### 3.1. Fungal Survey

**Figure 1:**
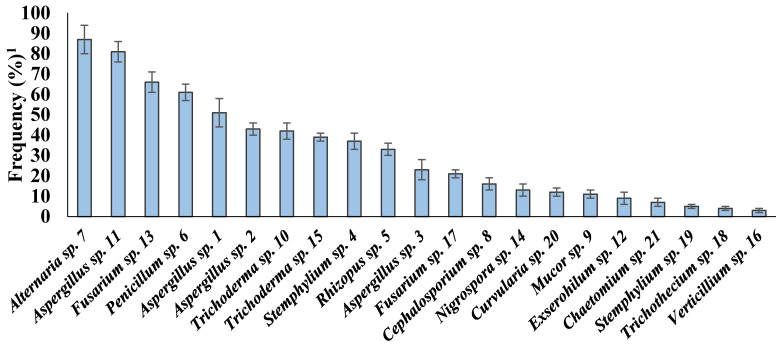
Frequency percentages of different fungi on maize stover (MS) (mean ± SD); ^1^ Frequency, % $=\frac{\mathrm{Number}\;\mathrm{of}\;\mathrm{infected}\;\mathrm{samples}}{\mathrm{Total}\;\mathrm{number}\;\mathrm{of}\;\mathrm{tested}\;\mathrm{samples}}\times 100.$

The isolation trial showed that plant residuals were associated with 21 species, belonging to 16 genera of fungi (Figure 1). All isolated fungi were identified to the genus level. All the genera were represented by one species except *Aspergillus*, which was represented by five species, and *Fusarium* by two species. Considerable differences in the frequency of the recorded fungi on MS of maize were observed. The highest frequently isolated fungi were *Alternaria* sp. 7 (87%), followed by *Aspergillus* sp. 11 (81%), *Fusarium* sp. 13 (66%), and *Penicillium* sp. 6 (61%). *Aspergillus* sp. 1 (51%), *Aspergillus* sp. 2 (43%), and *Trichoderma* sp. 10 (42%) were detected in a moderate amount. In contrast, *Verticillium* sp. 16 (3%), *Trichothecium* sp. 18 (4%), *Stemphylium* sp. 19 (5%), and *Chaetomium* sp. 21 (7%) were the lowest frequently recovered fungi.

### 3.2. Screening of Cellulolytic Activity and OA Production

**Figure 2:**
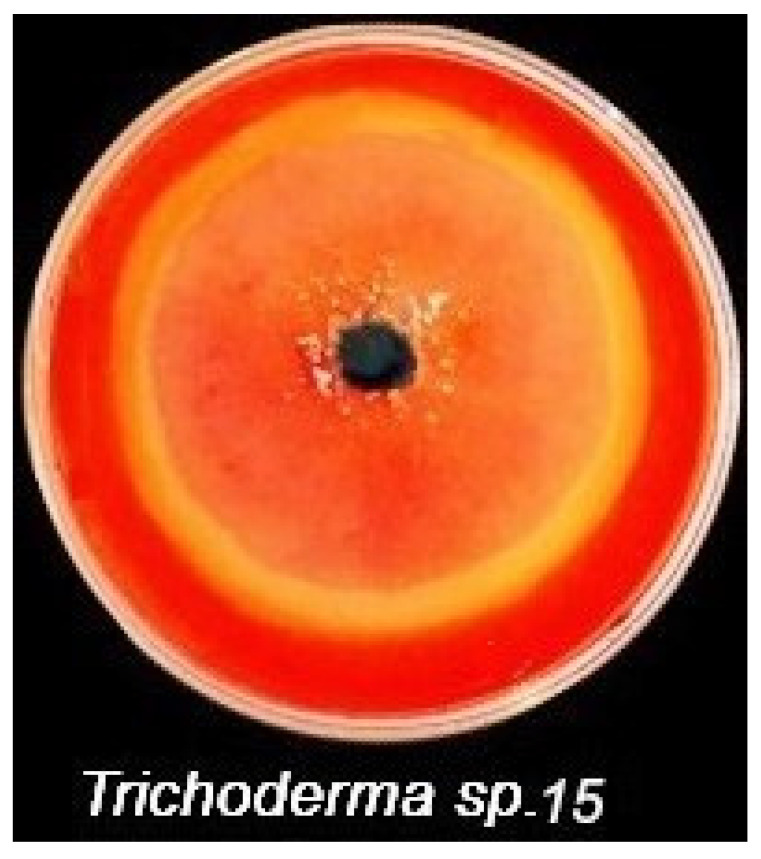
Plate assay of the active cellulose-degrading *Trichoderma* sp. 15 with halo degradation zone around the fungal growth.

To choose the most cellulolytic isolates, descriptive screening of the cellulolytic activity was performed on plates of agar medium containing Congo red dye. Out of the 21 isolates, 11 were able to develop a clear zone around the grown colony (Figure 2), reflecting the active cellulolytic of the tested fungi. Two isolates of *Trichoderma* sp. 10 and 15 were found to form the highest area of clear zone around the grown fungi.

Although the descriptive screening is quick and sheds some light on the overall information about a specific area under investigation, it is not enough to rely on it, because there are several aspects to be considered, especially when dealing with in-depth studies such as the present one. The next step was to verify the potentiality of the 11 fungal isolates to synthesis OA directly on the MS-based medium.

**Figure 3:**
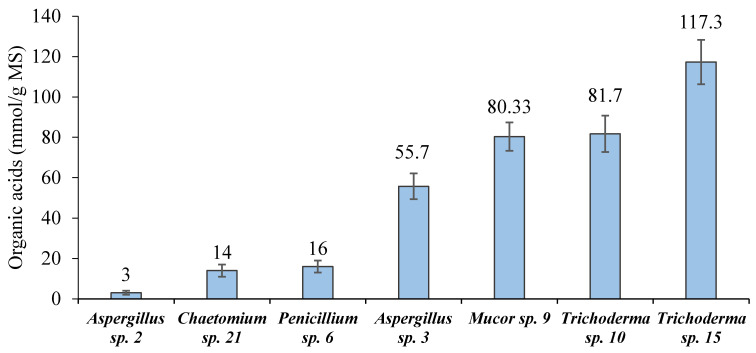
Screening of the cellulolytic fungal isolates for the biosynthesis of organic acids (mean ± SD).

As shown in Figure 3, out of the cellulolytic fungal isolates, seven isolates (2, 21, 6, 3, 9, 10 and 15, and 21 and 22) were found to be able to convert MS into OA. Of them, *Trichoderma* sp. 15 was superior (117.3 mmol/g MS). The full morphological identification up to the species level was carried out on the selected *Trichoderma* sp. 15, which was found to be *T. asperellum*.

### 3.3. HPLC Screening of OA in the Hydrolysate of MS

**Figure 4:**
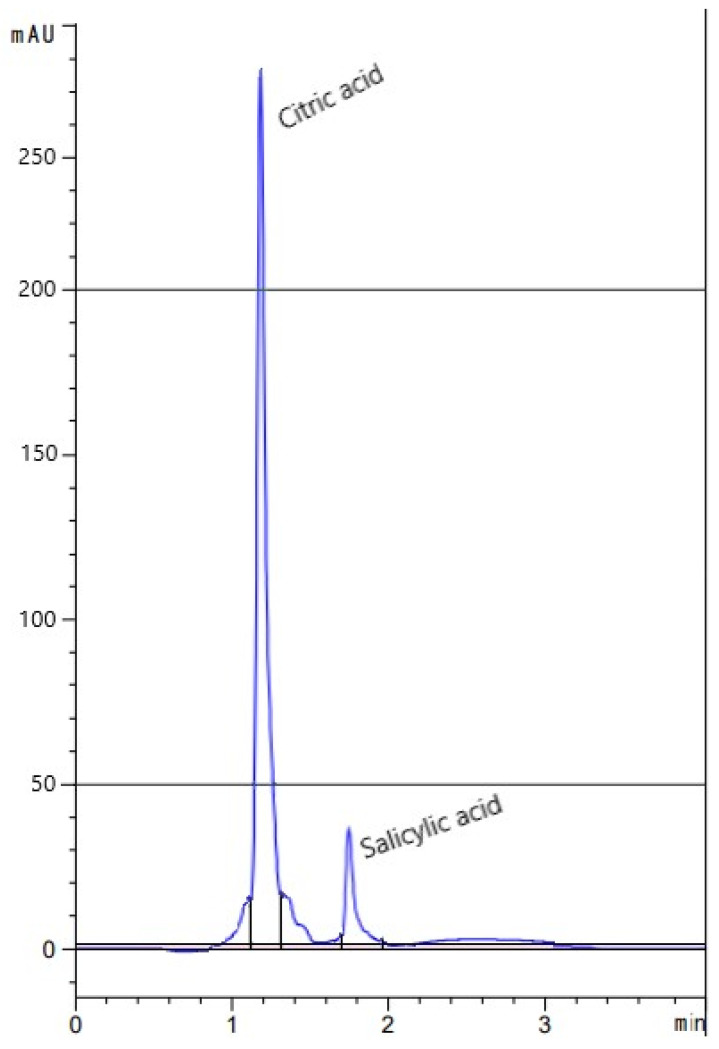
Chromatogram of organic acids in the hydrolysate of the fermented MS *Trichoderma* sp. 15, as detected by HPLC. *X*-axis: Retention time (min), *Y*-axis: Observed peak area (mAU = milli-absorbance units.

Based on the fermentation experiment, the scale-up fabrication of OA was performed on the MS-based medium. Following the fermentation, the hydrolysate of SSF of MS by the *Trichoderma* sp. 15 was screened, using the HPLC technique, for the various possible OA that may have been present. Given the simplicity, ease, accuracy, and rapidity of analysis, the HPLC technique is a desirable procedure. The result of HPLC is depicted in Figure 4.

Obviously, there were only two OAs detected in the post-culture filtrate of the 7-day-old fermented MS. The detected OA were citric (2.007 mg/g MS) and salicylic (0.193 mg/g MS) acids. Citric acid was generated in the greatest amount, representing about 91.2% of the two detected acids, whereas salicylic acid occupied only about 8.8%. Thus, this type of bio-fermentation could be considered a homofermentative, such that the entire or the majority of the end product was CA.

### 3.4. Molecular Identification’ of *Trichoderma* sp. 15

**Figure 5:**
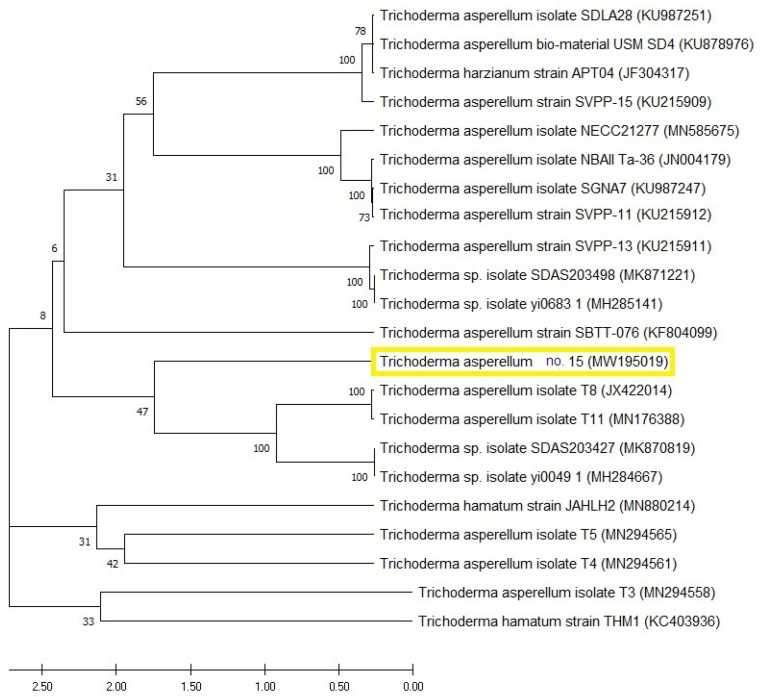
Molecular phylogenetic tree of the partial sequence of the internal transcribed spacer, showing the position of *Trichoderma* strain sp. 15 (highlighted with yellow color) with respect to the closely related sequences in GenBank.

The selected fungal strain (*Trichoderma* sp. 15) was characterized by the molecular technique of ITS as a perfect tool for identification. From Blast analysis, strain 15 displayed 99% similarity with the formerly identified *T. asperellum* on the Genbank. Figure 5 represents the constructed phylogenetic tree of *Trichoderma* sp. 15, which comes in line with the previous morphological identification. The GenBank accession number of the present fungal strain no. 15 (*T. asperellum*) was MW195019.

### 3.5. *T. asperellum* CA vs. *F. oxysporum*

**Figure 6:**
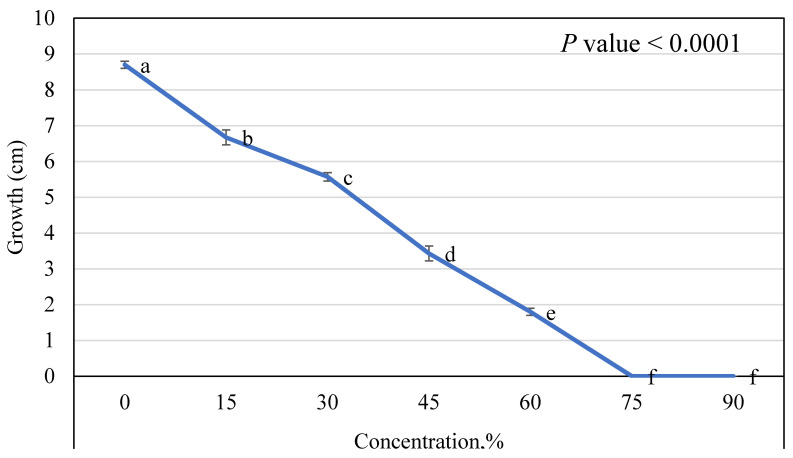
The growth of *F. oxysporum* was affected by different concentrations of CA of *T. asperellum* (mean ± SD). Different letter on a point indicates a significant difference at *p* ≤ 0.05.

In vitro, the antifungal effect of CA obtained from *T. asperellum* at different concentrations on the growth of FOL is depicted in Figure 6. Obtained data show that each of the tested CA concentrations had a strong inhibitory effect on the tested pathogen, even at the lower concentrations (15%). The inhibitory effect was increased when increasing CA concentrations. By increasing the CA concentration up to 75%, a complete inhibition in FOL growth was recorded.

### 3.6. *T. asperellum* CA vs. Germination

**Figure 7:**
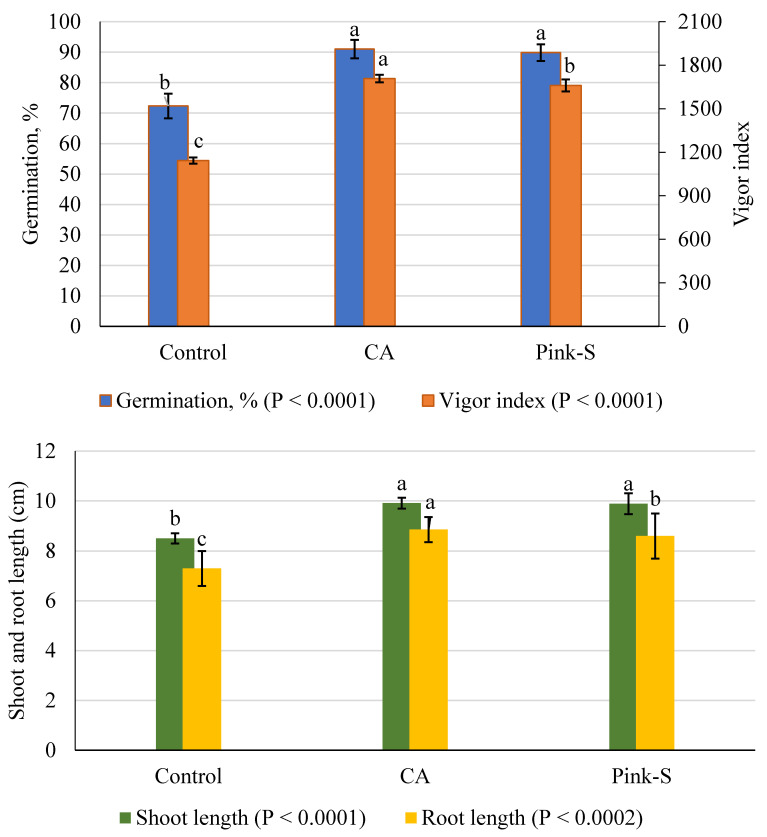
Influence of CA on germination and vigor index of tomato seeds (mean ± SD). For each criterion, the column designated with a different letter indicates significant differences (*p* ≤ 0.05).

The influence of crude CA on the germination of tomato seeds and seedling parameters was evaluated; the results are presented in Figure 7. The control treatment of germination was about 72.3%. However, CA was effective in improving the germination percentage (91%) with no significant difference with the chemical fungicide (89.83%) as compared to the control treatment. The same trend could be observed in the shoot length parameter. On the other hand, CA treatment came first, followed by fungicide application in improving root length parameter (8.85 and 8.59%, respectively) as compared to control (7.30%).

The vigor index was also affected by the application of CA, which increased the vigor index compared to the control and showed similar differences as in the other parameters. Seeds treated with CA showed a 49.40% increase of vigor index over the controls of tomato plants. The present data allow us to conclude that CA has the potentiality to enhance the germination and vigor index; this, in turn, is expected to reduce losses due to delayed germination. This treatment also showed the earliest highest seed germination (100%) at the fifth and sixth day compared to the control of both laboratory and field conditions, respectively.

### 3.7. Wilt Disease Management

**Figure 8:**
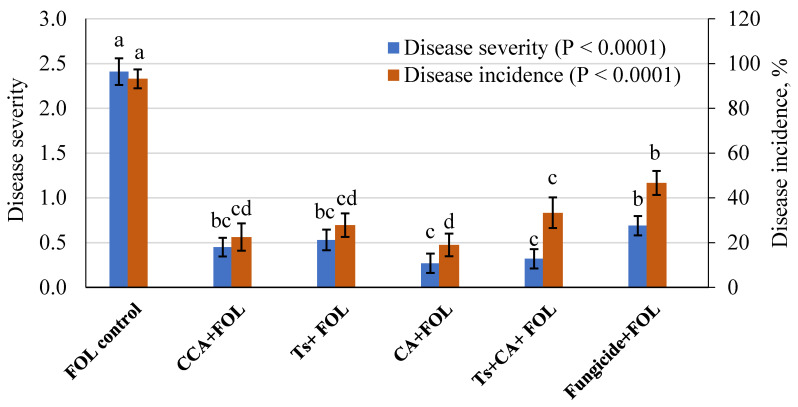
*T. asperellum* and its CA against wilt disease development of tomato seedlings after 40 days of sowing under greenhouse conditions (mean ± SD)**.** For each criterion, the column superscripted by the same letter(s) is not significantly different at *p* ≤ 0.05. FOL = *F. oxysporum* f, sp. *Lycopersici* pathogen, CCA = chemical citric acid, Ts = *T. asperellum* spores, CA = Citric acid of *Trichoderma*.

All the treatments appeared to markedly reduce the values of DS and DI compared to the untreated, infected control (Figure 8). In this respect, under FOL infection, the single application of CA treatment showed a higher decrease in DS and DI of tomato, being 0.27 and 19%, respectively. However, the single CCA treatment came in the second-order, followed by single Ts, then the combination of Ts + CA treatments. Fungicide treatment showed the lowest protection levels observed in DS and DI, being 0.69 and 46.67%, respectively.

### 3.8. Growth and Yield of Tomato

The data illustrated in Table 1 indicated that a significant variation was recorded for growth parameters of tomato plants in the absence or presence of FOL inoculum. Under infection stress with *Fusarium* wilt disease, a significant reduction in the yield parameters of the infected plants was recorded. However, under infection stress, an equal significant increment was observed in shoot and root length parameters due to single treatments with Ts, CA, its combinations, CCA, and the chemical fungicide as compared with infected control. Single Ts and Ts + CA combinations showed a significant increase in tomato leaves numbers. The same increment was recorded in fresh and dry weight plant^−1^ parameters due to single Ts, CA, or its combination treatments.

Without infection stress, a proportional increment in plant leaves number was recorded due to single Ts or combined Ts + CA treatments. CA treatment was effective in improving plant dry weight. The same significant increment was also recorded in plant yield due to CCA or combined Ts + CA applications as compared to the negative control.

Significant variation was recorded for yield parameters of tomato plants due to *Trichoderma* activity and CCA treatments compared to an inoculated control. In this respect, seedlings applications with combined Ts + CA showed the maximum significant increase in yield and weight of single fruit as compared to infected control. Single treatments with Ts ranked the second-best treatment, treatments with the CCA in increasing both yield parameters compared with the untreated infected control. However, fungicide and CA treatments showed the lowest significant increase in yield parameters. In the absence of FOL, there was no significant growth promotion response between any of the *Trichoderma* treatments and negative control plants.

### 3.9. Physiological Performance of Tomato Plants

#### 3.9.1. Pigments Content

Chlorophyll contents of tomato plants are presented in Table 2. In the presence of FOL, seedlings treated with CA treatments alleviated the harmful effect of the pathogen, in which the highest significant increase in total Chls (3.603, 3.739, 3.532, respectively) were recorded for single Ts and combined Ts + CA, followed by fungicide applications. Treatment of single CA and CCA came third in this respect in comparison to untreated infected control. However, the majority of treatments showed a significant increase in carotenoids contents compared to infected control. In the presence or absence of FOL infection, single CA and combined Ts + CA treatments presented the highest significant increase of carotenoids content as compared to their controls.

#### 3.9.2. Total Phenol and Defense-Related Enzymes

Total phenol, PPO, and POD were determined in the plants (Table 3) since they play an important role in plant protection. The obtained results indicated that under infection by FOL, the application of Ts + CA and CCA treatments led to a significant increase in the total phenolic contents in tomato shoots. Among all treatments, CCA + FOL showed a highly significant increase in peroxidase activity, followed by Ts alone compared with infected control. However, seedlings treatment with single Ts showed the maximum significant increase in polyphenol oxidase activity as compared to infected control.

## 4. Discussion

The relative diversity of the cellulose-decomposing fungi isolated from MS may be due to the simple isolation method through using decayed MS samples themselves as a source for the isolation of fungi and, at the same time, as the only nutritive source for growth fungal, which is more similar to what happens in nature. The fermentation conditions, like water activity, type of substrate, temperature, and air gas composition, are the greatest determinant parameters, affecting the growth and development of the fungal microbiome in nature [34]. For instance, when water activity is a limiting issue during the plant growth in the soil, *Fusarium* sp. is common and can continue to grow in a limited form under the storage with appropriate moisture [35]. On the contrary, *Aspergillus* spp. and *Penicillium* spp. were found to be more xerophilic and could get the upper hand during storage. However, after harvesting, these fungi colonize the plant under storage; e.g., *A. flavus* is found in the soil associated with the plant material, which occurs in developing kernels in the field and later multiplies in storage if conditions permit. Moreover, these fungi were reported to be mycotoxigenic, representing possible health hazards of the contaminated products. On the other hand, it may affect seed quality, germination, viability, seedling vigor, and root development [33.34].

The MS-based fermentation medium was supplemented by a complex inorganic form of phosphorus. TCP induces fungi to produce OA. The capacity to solubilize complex phosphates is mainly attributed to the synthesis of OA, which is produced through the cellulose-decomposition [9,15]. An earlier study stated a significant correlation between the gradual decrease in pH and the release of OA [36].

The primary structure of MS, which was utilized during the fermentation process, is hard to degrade since it is composed mainly of lignin, cellulose, and hemicellulose; such complicated construction minimizes the biodegradability into monomers, so the bioremediation process into monomers needs the cooperation of various enzymes. The main targeted component is cellulose, which degrades into single units of D-glucose, which is a fermentable sugar for most microorganisms [37]. Cellulase enzymes catalyze the breakdown of cellulose into its monomers (glucose). Among cellulases, endoglucanase randomly cleaves the β-1,4-glucosidic bonds in the inner part of cellulose, representing the intermediate step of hydrolysis, which, in combination with the catalytic action of the other cellulases, liberates the monomers of glucose [38]. By the same token, xylan is hydrolyzed by the combined action of endo-1,4-β-xylanase and β-D-xylosidases, releasing fermentable monomer of D-xylose [39]. These monomers are the starting point and/or the cornerstone for the biosynthesis of various molecules, including OA, by the microorganism [37,38,39].

Indeed, *A. oryzae* and *A. niger* were distinguished by the bio-production of a vast amount of OA in the existence of a complex form of phosphorus such as rock phosphate; such OA have been also recognized for phosphate solubilization by various species of *Penicillium,* e.g., *P. chrysogenum* [36,40]. Moreover, the nature and kind of the produced-OA have a considerable impact on the solubilization of complex phosphates; i.e., the amount of released P is mediated by both the type and position of the functional group within each acid [41]. As far as we know, little knowledge is available about the types and amount of OA produced by the *Trichoderma*. Therefore, *Trichoderma* sp. 15 was chosen for further studies, as the most active cellulolytic and potent producer of OA in general and CA in particular in the current investigation. Although microbial bio-production of CA is commercially well defined and reported by microorganisms including *Trichoderma* spp. [42], there is not enough information concerning the amount and kind of OA generated during the bioprocessing of plant residues. Therefore, this study shed some light on this region of search. Microbial degradation is the preliminary action in such a process, followed by individual biochemical conversion to every target molecule [36]. For instance, although citric and oxalic acids were the main acidic metabolites of fungal fermentation, each one has a different formation route. CA is created under nitrogen-limited situations, while oxalic acid is induced under carbon-limited conditions [42]. Salicylic acid is formed from cinnamic acid by (1) decarboxylation of cinnamic acid to benzoic acid and then hydroxylation to salicylic acid, or (2) hydroxylation of cinnamic acid to *o*-coumaric acid that decarboxylated to salicylic acid [43]. That is, each acid has a distinctive pathway that differs for each. On the other hand, some acids have parallel routes, like citrate and oxalate, whose fermentation is only one enzymatic step from the primary metabolism of D-glucose and D-fructose, respectively; another, the malate production mechanism, is shortened by one step of the fumarate pathway [44].

Concerning the fermentation conditions, OA could putatively be formed under carbon- and nitrogen-limiting conditions such as in the case of the present MS-based fermentation medium. In this case, variability in the composition of MS due to seasonal climatic changes must be taken into account, although these changes vary in a narrow range [36]. Complex phosphate is also a determinant factor for OA biosynthesis, where microbial solubilization of, e.g., rock phosphate is frequently attributed to the production of OA; the latter is also reported as an important product of cellulosic bioconversion by fungi [41]. Following the culture conditions reported above, the production of CA by *Trichoderma* sp. 15 in SSF was scaled up, and the filtrate was used as a source of crude CA.

The selected fungus was morphologically and molecularly identified as *T. asperellum*. Because of high sensitivity and specificity, molecular identification is widely used for the rapid identification of filamentous fungi at various taxonomic levels. The technique is set up for the comparison of the sequence coding for 18S rRNA gene after PCR amplification, using ITS, whose fragment size is uniform in numerous groups of fungi, making nucleotide sequencing of ITS fractions a prerequisite for revealing interspecific and, in some cases, intraspecific variation [45]. The constructed phylogeny, based on the well-identified sequence, is completely annotated and shows a tight correlation with those of similar strains. The ITS region could be used in barcode identification for different fungi, especially Basidiomycota [46]. Moreover, the ITS region is usually used and could be sufficient for fungal identification on the species level [47]. The ITS region is also considered to be among the markers with the fastest and highest probability of correct identifications for a very broad group of fungi [48]. Harmonically, the molecular identification came in line with the morphological one.

There was a positive effect of crude CA on seed germination and seedling features. The secondary metabolites produced by *Trichoderma* species were reported to contain growth stimulators like gibberellic acid and indole acetic acid [9,12,49], which possibly act as auxin-like molecules. On the other hand, the production of vigorous and healthy seedlings may be attributed to the interaction between crude CA of *Trichoderma* and induced overall tolerance in tomato seedlings toward seed-borne pathogens. In this respect, Li [10] reported an increase in dry matter accumulation in tomato roots and shoots of seedlings and also made seedlings longer and caused branched roots due to *Trichoderma* application; this resembles the main factor increasing seedlings’ transplantation success.

The disease parameters and the pathogenic fungus were decreased in the presence of *T. asperellum* and/or its CA. These results may be attributed to the strong capacity of *Trichoderma* sp. to solubilize and uptake nutrients from the soil, which makes it more competitive and effective than the other soil microbes [50]. Moreover, besides containing crude CA, the filtrate of *T. asperellum* could contain other antifungal constitutes such as aliphatic hydrocarbon compounds, terpenes, and fatty acids [51]. This process could be related also to the production of CA, which decreases the pH within a range that does not favor the growth of the pathogen, reducing the cytoplasmic pH and stopping metabolic activities. On the other hand, OA acts on the neutralization of the electrochemical potential and increases the permeability of the plasmic membrane, leading to the death of the pathogen [15,20,52]. In the many studies about the antimicrobial effect of CA, it was reported that CA possesses a stronger inhibitory impact compared to lactic and acetic acids [42].

The metabolites of fungal microbiota primarily affect the seed quality, germination, viability, seedling vigor, and the growth of root and coleoptile [35]. In this respect, Islam [53] reported the positive potentiality of five *Trichoderma* strains on enhancing the germination percentage and vigor index.

Similarly, Kaveh [54] reported a significant effect of *Trichoderma harzianum* Bi on the enhancement of seed germination, seedling quality, and field establishment of two muskmelon cultivars at the greenhouse and field conditions. Recently, Singh [55] found that among six vegetable crops, biopriming seeds of tomato with *T. asperellum* spores was effective for enhancement of seed germination and radicle length, which additionally triggered the various defense responses such as high phenylpropanoid activities and lignification in bio-primed tomato seedlings compared to the control. The enhancement of the speed of germination and seedling vigor of tomato seeds by *Trichoderma* spp. has been reported; moreover, seed and/or soil treatment with *Trichoderma* enhances seed germination percentage, directly by activating enzymes and phytohormones and indirectly by altering soil microbiota and nutrient availability in soil [49]. Additionally, similar to the CA produced by the current *T. asperellum*, the application of CA was reported to enhance castor beans growth [51].

*Trichoderma* spp. can stimulate growth and protect plants against various phytopathogens through their secondary metabolites [12]. The ability to stimulate plant development is mediated by the activation of auxin-dependent mechanisms and/or producing auxin analogs [9] such as Koninginins, 6PP, trichocaranes A-D, harzianolide, harzianopyridone, cyclonerodiol, and harzianic acid affect plant growth [12]. In this respect, Li [10] reported the *T*. *asperellum* strain CHF 78 with several plant growth-promoting abilities, including solubilization of complex phosphates such as Ca_3_(PO_4_)_2_ and production of cellulases, proteases, chitinases, indole acetic acid, and siderophores. Furthermore, this strain significantly increased the growth features of tomato plants infected or not infected with FOL.

Generally, OA and citric acid in particular were stated as antimicrobial agents [42]. *T. asperellum* has been reported as a mycoparasitic species that is well known and widely used for its ability to inhibit the growth of plant pathogens as well as protection of vegetables and other crops [9,56]. Their multi-enzymatic systems and anti-microbial capability make them one of the best alternative strategies for pesticides for tomato wilt management caused by FOL [10]. A positive correlation between the antagonistic capacity of *T. asperellum* in the form of chitinase and β-1,3-glucanase activities towards FOL was reported [8,10].

*Trichoderma* spp. can suppress the pathogen by the direct antagonism against the fungal pathogen and the production of CA and the secretion of hydrolytic enzymes (e.g., β-1,3-glucanases and chitinases), which degrade the fungal cell wall, thereby limiting the growth of the pathogen [8,42]. Therefore, environmentally friendly products of *Trichoderma* have commercially been used for plant protection and yield maximization [13].

Regarding the pigmentation status of the plant, Toma’nkova [57] reported that the reduction in the chlorophyll a/b ratio in plants infected by the FOL pathogen could occur mainly because of the decrease in Chla content. Carotenoid levels also presented a significant reduction in the case of FOL pathogen control. This result may due to the expansion of the infection that accompanied cell death as a result of the damage that occurred due to the pathogen in leaf tissue. Conversely, the presence of CA was found to enhance the photosynthesis process and alleviate the oxidative stress in castor beans [51].

The increment in chlorophyll content is a good indicator of crop health and productivity and boosts the efficiency of photosynthetic apparatus with an improved potential for stress resistance [51]. In this respect, apocarotenoids, a carotenoid-derived molecule, which includes isoprenoids, was reported to have an important role in plant–environment interfaces such as the defense against pathogens, attraction of pollinators (chemo-attractants), repellents by the volatile aromatic compounds, and growth regulators (inhibitors and stimulators, such as abscisic acid, and strigolactones) [58]. Moreover, the ability of *Trichoderma* spp. to improve several physiological processes, including stomatal conductance, net photosynthetic rate, transpiration, water use efficiency, internal CO_2_ concentration, and nutrient uptake, was reported [49]. Another study reported that *T. asperellum* confers tomatoes protection by inducing the expression of defense-related genes (chitinase and β-1,3-glucanase) in tomato plants against the *Fusarium* wilt pathogen [59].

Plants’ defense mechanisms start with a fast aggregation of phenols at the location of the infection, which constrain the progress of the pathogen since phenols act as an antimicrobial, antioxidant, and photoreceptor [60]. There are various defense mechanisms of PPO, including (1) broad-spectrum toxicity of PPO-generated quinones against the invasion pathogens, (2) alkylation and reducing the availability of cellular proteins to the pathogen, (3) creating a physical wall against the pathogens in the cell wall by crosslinking of quinones with protein or other phenolics, and (4) cycling of quinone redox, leading to the generation of H_2_O_2_ and other reactive oxygen species, which are recognized to be serious factors in plant-pathogen interaction and defense signaling [61].

The variation in POD activity in the infected plants may be due to the presence of a fast response by plants that augments the defense-related enzymes 5-folds on the first day, then starts to decrease by the 6th day, as a result of pathogen multiplication [62]. Another explanation suggested that POD is continuously active in plants at all times at various degrees due to the natural dynamics such as rain, wind, and gravity as well as the degree of exposure of plants to such mechanical dynamics such as chronic winds of varying intensity [63].

Collectively, POD can play a critical role in the integrated defense response of plants to an assortment of stresses, including cell wall toughening, acting as a toxic secondary metabolite, and stimulation of antioxidant capabilities [62,63].

## 5. Conclusions

Ultimately, the antagonism of *T. asperellum* against Fusarium wilt disease of the tomato might be supported in a multivariate model, such as the production of CA, anti-fusarium agents, and/or producing growth-promoting substances. This study shed some light on the production of the bioactive compound CA from MS-based medium, using *T. asperellum*; the resulting crude CA was successfully applied as a biological control agent. The potential responses in tomato growth, yield, and biochemical parameters are promising. Because of the success of the production of such bioactive compounds from MS, it could be recommended to be valid in the management of plant diseases, especially the Fusarium wilt disease of the tomato. However, additional research is proposed for maximization of the production process and separation of CA in pure crystals.

## Figures and Tables

**Table 1:** Growth parameters and yield of tomato as affected by different treatments of fungal CA under greenhouse conditions (mean ± SD).

Treatment	Shoot Length (cm)	Root Length (cm)	Leaves No. Plant^−1^	Plant Fresh Weight (g)	Plant Dry Weight (g)	Plant Yield (g)	Plant Fruit Weight (g)	Fruits No. Plant^−1^
Control	49.8 ± 5.9 ab	9.20 ± 1.8 a	7.0 ± 1.0 bc	8.82 ± 0.8 ab	1.92 ± 0.1 c	648.1 ± 28.7 bc	55.50 ± 3.1 a	13.00 ± 1.0 abc
FOL control	39.7 ± 7.9 c	7.30 ± 0.3 b	6.2 ± 0.8 c	5.18 ± 0.7 c	1.10 ± 0.2 d	283.1 ± 15.6 g	32.00 ± 0.7 d	10.00 ± 1.0 f
Fungicide + FOL	47.2 ± 6.1 ab	9.20 ± 0.8 a	7.0 ± 1.2 bc	7.12 ± 1.1 b	1.94 ± 0.3 c	410.2 ± 16.7 f	35.83 ± 1.7 cd	11.00 ± 0.1 ef
CCA + FOL	52.9 ± 6.4 ab	9.00 ± 0.7 a	7.0 ± 0.7 bc	7.60 ± 0.5 b	2.02 ± 0.2 bc	476.1 ± 4.0 e	44.17 ± 1.4 b	11.67 ± 0.6 cde
Ts + FOL	51.8 ± 6.1 ab	9.70 ± 1.8 a	7.8 ± 1.3 ab	8.74 ± 2.2 ab	2.10 ± 0.5 bc	449.7 ± 22.5 e	32.92 ± 4.0 d	12.33 ± 0.6 bcde
CA + FOL	55.1 ± 8.4 a	9.40 ± 1.1 a	7.2 ± 1.1 bc	8.88 ± 2.4 ab	2.16 ± 0.5 abc	404.2 ± 7.0 f	38.39 ± 6.4 c	11.33 ± 0.6 def
Ts + CA + FOL	51.9 ± 3.4 ab	9.70 ± 1.0 a	8.4 ± 0.5 a	8.36 ± 1.5 ab	2.24 ± 0.1 abc	560.0 ± 17.4 d	45.99 ± 1.9 b	12.67 ± 0.7 bcd
CCA	54.7 ± 2.5 ab	10.1 ± 1.7 a	8.0 ± 0.7 ab	9.56 ± 0.8 a	2.42 ± 0.4 ab	679.3 ± 18.5 a	55.21 ± 2.8 a	13.67 ± 0.6 ab
Ts	50.6 ± 3.6 ab	9.90 ± 1.3 a	8.4 ± 0.5 a	9.96 ± 0.8 a	2.24 ± 0.3 abc	663.6 ± 20.4 ab	53.63 ± 3.6 a	13.33 ± 1.5 ab
CA	46.5 ± 1.9 bc	9.60 ± 1.1 a	7.8 ± 0.4 ab	8.54 ± 0.7 ab	2.14 ± 0.3 abc	633.9 ± 5.5 c	51.90 ± 0.6 a	13.33 ± 1.5 ab
Ts + CA	53.8 ± 4.8 ab	10.4 ± 0.5 a	8.4 ± 0.5 a	9.60 ± 0.5 a	2.54 ± 0.2 a	689.2 ± 40.1 a	55.13 ± 2.2 a	14.33 ± 0.6 a
*p* value	=0.0035	=0.0365	=0.0008	<0.0001	<0.0001	<0.0001	<0.0001	<0.0001

FOL = *F. oxysporum* f, sp. *Lycopersici* pathogen, CCA = chemical citric acid, Ts = *T. asperellum* 15 spores, CA = citric acid of *Trichoderma*. Means within the column followed by the same letter(s) are not significantly differed at *p* ≤ 0.05.

**Table 2:** Chlorophyll contents of tomato plants as affected by the inoculation by *T. asperellum* and CA treatments under greenhouse conditions (mean ± SD).

Treatment	Chlorophyll Contents (mg/g Fresh Weight)			
	Chla	Chlb	Total Chls	Carotenoid
Control	2.698 ± 0.07 bc	1.186 ± 0.07 bc	3.884 ± 0.07 cd	0.820 ± 0.01 c
FOL control	1.676 ± 0.19 d	0.494 ± 0.19 e	2.170 ± 0.18 h	0.628 ± 0.07 d
Fungicide + FOL	2.712 ± 0.01 bc	0.821 ± 0.01 d	3.532 ± 0.10 f	0.883 ± 0.01 bc
CCA + FOL	2.570 ± 0.12 c	0.796 ± 0.12 d	3.366 ± 0.05 g	0.856 ± 0.03 c
Ts + FOL	2.662 ± 0.29 bc	0.941 ± 0.29 cd	3.603 ± 0.10 ef	0.845 ± 0.18 c
CA + FOL	2.884 ± 0.30 b	0.379 ± 0.30 e	3.263 ± 0.19 g	1.020 ± 0.14 a
Ts + CA + FOL	2.758 ± 0.03 bc	0.981 ± 0.03 cd	3.739 ± 0.03 de	0.915 ± 0.05 abc
CCA	2.682 ± 0.08 bc	1.303 ± 0.08 ab	3.985 ± 0.01 bc	0.619 ± 0.01 d
Ts	2.597 ± 0.06 bc	1.508 ± 0.06 a	4.104 ± 0.02 b	0.808 ± 0.01 c
CA	2.869 ± 0.0.3 b	0.949 ± 0.03 cd	3.818 ± 0.01 d	0.918 ± 0.01 abc
Ts + CA	3.280 ± 0.01 a	1.235 ± 0.01 abc	4.515 ± 0.02 a	1.009 ± 0.02 ab
*p* value	<0.0001	<0.0001	<0.0001	<0.0001

FOL = *F. oxysporum* f, sp. *Lycopersici* pathogen, CCA = chemical citric acid, Ts = *T. asperellum* spores, CA = citric acid of *Trichoderma*. The same letter(s) within a column indicates the non-significant difference (*p* ≤ 0.05).

**Table 3:** Biochemical activities of tomato plants as affected by the inoculation by *T. asperellum* and CA treatments under greenhouse conditions (mean ± SD).

Treatment	Total Phenol (mg Catechol 100 g^−1^)	Enzyme (U g^−1^ Fresh wt.)	
		Peroxidase	Polyphenol Oxidase
Control	27.48 ± 1.50 ab	28.77 ± 1.57 d	18.33 ± 2.08 g
FOL control	16.67 ± 2.08 f	14.67 ± 1.53 h	10.00 ± 2.00 h
Fungicide + FOL	19.75 ± 1.39 de	11.00 ± 1.00 i	17.33 ± 1.53 g
CCA + FOL	26.87 ± 1.21 ab	49.43 ± 2.89 a	20.90 ± 2.82 f
Ts + FOL	23.30 ± 1.47 c	16.67 ± 1.53 gh	11.00 ± 2.00 h
CA + FOL	19.50 ± 1.80 de	34.67 ± 2.52 c	32.67 ± 2.52 c
Ts + CA + FOL	29.37 ± 2.03 a	18.33 ± 2.08 fg	27.00 ± 2.65 e
CCA	22.07 ± 0.90 cd	11.33 ± 2.08 i	20.00 ± 2.00 f
Ts	27.90 ± 1.15 ab	40.00 ± 2.00 b	45.77 ± 2.36 a
CA	18.23 ± 1.37 ef	20.23 ± 1.66 ef	31.10 ± 1.85 d
Ts + CA	26.20 ± 0.72 b	21.30 ± 2.14 e	39.43 ± 1.91 b
*p* value	< 0.0001	<0.0001	<0.0001

FOL = *F. oxysporum* f, sp. *Lycopersici* pathogen, CCA = chemical citric acid, Ts = *T. asperellum* spores, CA = citric acid of *Trichoderma*. The same letter(s) within a column indicates the non-significant difference (*p* ≤ 0.05).

## Data Availability

Relevant data applicable to this research are within the paper.
